# The nonlinear relationship between the hepatic steatosis index and hypertension: NHANES 2017–2020

**DOI:** 10.1097/MD.0000000000045755

**Published:** 2025-11-07

**Authors:** Zihao Zhao, Yuhong Ma, Weizhong Huangfu

**Affiliations:** a Department of General Practice, The Affiliated Hospital of Inner Mongolia Medical University, Hohhot, China.

**Keywords:** hepatic steatosis index, hypertension, nonalcoholic fatty liver disease, nonlinear relationship

## Abstract

The hepatic steatosis index (HSI), a noninvasive tool for assessing hepatic steatosis, has been linked to hypertension. This study aimed to investigate their nonlinear relationship and modifying factors. Using 2017–2020 NHANES data, we analyzed 7723 adults aged ≥ 20 years. Pearson chi-square test and Student *t* test were used to compare baseline characteristics across HSI quartiles (Table 1). Multivariable logistic regression assessed HSI-hypertension associations, while threshold regression identified inflection points. Subgroup analyses evaluated effect modifications. Baseline Characteristics: Higher HSI quartile (Q4) had a significantly different age distribution (*P* < .001), with the highest quartile (Q4) being younger on average (48.34 ± 16.28 years) than Q2 and Q3, female predominance (66.25%), and lower socioeconomic status (income-to-poverty ratio 2.38 ± 1.47, *P* < .001). Metabolic comorbidities showed dose-response trends, with diabetes prevalence rising from 5.33% (Q1) to 23.08% (Q4) (*P* < .001). Primary analysis: Each HSI unit was associated with a 4% higher risk of hypertension (odds ratio [OR] = 1.04, 95% confidence interval [CI]: 1.03–1.05). Q4 had a 2.65-fold higher risk than Q1 (95% CI: 2.23–3.15). Nonlinearity: A significant inflection point emerged at HSI = 50.33, with steeper risk slope below threshold (OR = 1.06, *P* < .001) versus null association above (OR = 0.99, *P* = .181). Subgroup Heterogeneity: Stronger associations occurred in males (β = 1.06 vs females 1.02), older adults (≥60 years), and coronary heart disease patients (threshold lowered to 38.67) (P interaction < 0.05). HSI exhibits a threshold-based nonlinear association with hypertension, demonstrating stronger associations in males, older adults, and cardiovascular disease subgroups, supporting its utility as a marker in metabolic hypertension risk stratification.

## 1. Introduction

Nonalcoholic fatty liver disease (NAFLD) has become the most common chronic liver disease worldwide, affecting approximately 25% of the adult population.^[1]^ The core feature of NAFLD is the excessive accumulation of fat within liver cells (without a history of heavy alcohol consumption or other secondary causes).^[2]^ Its pathological spectrum ranges from benign simple fatty degeneration to progressive nonalcoholic steatohepatitis, liver fibrosis, cirrhosis, and even hepatocellular carcinoma.^[3]^ In addition to its direct impact on liver-related morbidity and mortality, NAFLD is increasingly regarded as a systemic metabolic disorder disease, closely related to a series of cardiovascular risk factors such as obesity, insulin resistance, hyperlipidemia, and hypertension.^[4]^

Among these complications, hypertension is one of the major health burdens worldwide: the number of affected adults globally exceeds 1.3 billion, ranking as the third leading cause of death globally, and it is also the primary trigger for stroke, coronary heart disease and heart-kidney failure.^[5–7]^ Increasing evidence indicates that NAFLD is an independent risk factor for hypertension, with approximately 50% of hypertensive patients having liver steatosis.^[8,9]^ From a mechanistic perspective, this association is believed to be related to chronic inflammation activation, oxidative stress cascade reactions, endothelial dysfunction, and dysregulation of fat factor secretion, which link liver fat accumulation to the disruption of blood pressure homeostasis.^[10,11]^ However, previous studies exploring the association between NAFLD and hypertension mostly relied on invasive methods (such as liver biopsy) as diagnostic tools for NAFLD, which limited their application in clinical practice and population studies.^[12]^

To enhance clinical management of NAFLD, researchers have recently developed the hepatic steatosis index (HSI) as an innovative screening tool. Calculated through logistic regression modeling using the formula: HSI = 8 × (alanine aminotransferase/aspartate aminotransferase ratio) + body mass index (+2 for females; +2 for diabetes comorbidity),^[13]^ this index combines non-invasiveness, simplicity, and diagnostic efficacy. While HSI demonstrates strong validity in assessing hepatic steatosis, emerging evidence also reveals its associations with type 2 diabetes, cardiovascular diseases, and hypertension, suggesting its potential as a multidimensional predictor of metabolic disorders.^[14–16]^ Nevertheless, comprehensive investigations that characterize the quantitative association between HSI and hypertension risk, particularly its shape or pattern (e.g., linearity vs nonlinearity), remain limited. To address this gap, we systematically examined the nonlinear association relationship between HSI and hypertension using data from the 2017–2020 National Health and Nutrition Examination Survey (NHANES), providing evidence-based insights for early prevention of metabolic hypertension.

## 2. Methods

### 2.1. Study design and population

The data utilized in this study were derived from the 2017–2020 cycles of the NHANES, an ongoing, cross-sectional surveillance program conducted by the Centers for Disease Control and Prevention. NHANES employs a complex, multistage, stratified probability sampling design to select a nationally representative sample of non-institutionalized U.S. residents, with the primary objective of assessing population health trends and nutritional status across all age groups. The study protocol received ethical approval from the National Center for Health Statistics Research Ethics Review Board. Written informed consent was obtained from all adult participants and legally authorized representatives of minors prior to data collection. Comprehensive documentation regarding the survey methodology and ethical considerations is accessible through the official NHANES website at https://www.cdc.gov/nchs/nhanes/about/erb.html?CDC_AAref_Val=https://www.cdc.gov/nchs/nhanes/irba98.htm.

**Figure 1.**
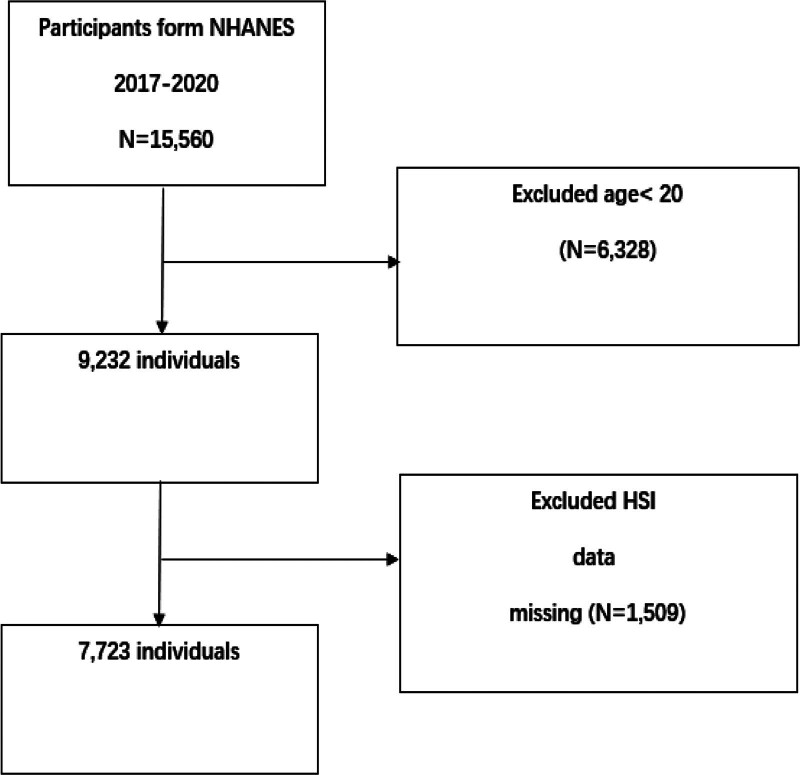
Flowchart of participant selection in NHANES 2017–2020.

This cross-sectional study utilized data from the 2017–2020 cycle of the NHANES, initially involving 15,560 participants. The exclusion criteria were as follows: individuals younger than 20 years (n = 6328) and those with missing HSI measurements (n = 1509). After applying these exclusions, the final analytical cohort comprised 7723 eligible participants (Fig. 1).

### 2.2. Exposure and outcome variables

#### 2.2.1. HSI

HSI was calculated as follows: 8 × (alanine aminotransferase/aspartate aminotransferase ratio) +  body mass index (+ 2, if female; +2, if diabetes mellitus). Diabetes mellitus was defined by self-reported physician diagnosis (yes/no), ascertained through the questionnaire item: “Other than during pregnancy, has a doctor ever told you that you have diabetes?.”

#### 2.2.2. Hypertension

Hypertension case ascertainment was operationalized through structured questionnaire responses. Participants were categorized as hypertensive if they provided affirmative responses to the item: “Has a doctor or health professional ever told you that you have hypertension?” This diagnostic classification aligns with the criteria established in the Seventh Report of the Joint National Committee on Prevention, Detection, Evaluation, and Treatment of High Blood Pressure (JNC 7).^[17]^

#### 2.2.3. Covariates

We included several covariates that could potentially influence the association between HSI and hypertension. These covariates comprised: sociodemographic characteristics, age, gender, race/ethnicity, educational attainment, income-to-poverty ratio, and marital status; lifestyle factors, alcohol consumption status and smoking status; and self-reported medical histories including coronary heart disease, congestive heart failure, hyperlipidemia, diabetes mellitus, and kidney stones. Smoking status was categorized as current smoker or non-current smoker. Current smokers were defined as participants who answered “yes” to the question “Do you now smoke cigarettes?” (NHANES variable: SMQ040). Non-current smokers included former smokers and never smokers. Alcohol consumption status was categorized as current drinker or non-current drinker (abstainer). Current drinkers were defined as participants who reported consuming at least one alcoholic beverage in the past 12 months (NHANES variable: ALQ101). Non-current drinkers reported no alcohol consumption in the past 12 months.

### 2.3. Statistical analysis

Statistical analyses were performed using R software (version 4.2.0; R Foundation for Statistical Computing). Statistical significance was determined at a 2-tailed α level of 0.05. For continuous variables, 1-way analysis of variance was used to compare differences among 3 or more groups (e.g., baseline characteristics across HSI quartiles) if data were normally distributed; the Kruskal-Wallis test was applied for non-normally distributed continuous variables. Student *t* test was used for comparisons between 2 groups. Categorical variables were compared using Pearson chi-square test.

Three multivariable logistic regression models were constructed to evaluate the association between the HSI and hypertension risk: Model 1 (unadjusted); Model 2 adjusted for age, sex, and race/ethnicity; and Model 3 with additional adjustments for socioeconomic status (poverty-income ratio, education level, marital status), lifestyle factors (alcohol consumption, smoking status), and comorbidities (hypercholesterolemia, diabetes mellitus, kidney stones, coronary heart disease, and congestive heart failure). Results were expressed as odds ratios (ORs) with 95% confidence intervals (CIs).

Spline smoothing methods were employed to examine potential nonlinear relationships between the HSI and hypertension. Stratified analyses and interaction tests were conducted to assess potential effect modifications across subgroups.

## 3. Results

### 3.1. Baseline characteristics

The baseline characteristics of participants across HSI quartiles are presented in Table 1. Significant differences (*P* < .05) were observed in most variables except alcohol consumption (*P* = .117), indicating substantial heterogeneity in sociodemographic and clinical profiles among HSI subgroups. Notably, the highest HSI quartile (Q4) exhibited younger mean age (48.34 ± 16.28 vs 50.11 ± 19.03 in Q1, *P* < .001) and a predominance of females (66.25% vs 31.59% in Q1). Racial disparities were prominent, with Non-Hispanic Black individuals comprising 30.23% of Q4 compared to 25.01% in Q1. Lower socioeconomic status was associated with higher HSI levels, evidenced by reduced income-to-poverty ratios (Q4: 2.38 ± 1.47 vs Q1: 2.57 ± 1.51, *P* < .001) and higher proportions of individuals with less than high school education (Q4: 42.18% vs Q1: 43.24%, *P* < .001). Metabolic comorbidities demonstrated a dose-response relationship with HSI quartiles. Diabetes prevalence increased markedly from 5.33% in Q1 to 23.08% in Q4 (*P* < .001), while hypercholesterolemia peaked in Q3 (40.57%) before declining in Q4 (35.71%).Despite nonsignificant differences in alcohol consumption (*P* = .117), smoking rates showed a U-shaped association, with the lowest prevalence in Q2 (39.90%) and Q3 (38.96%), rising to 41.30% in Q4 (*P* < .001) (Table 1).

**Table 1:** Baseline characteristics of participants.

Quartiles of HSI characteristic	Q1 (n = 1931)	Q2 (n = 1930)	Q3 (n = 1930)	Q4 (n = 1932)	*P*-value
Age	50.11 ± 19.03	52.83 ± 16.92	52.20 ± 17.01	48.34 ± 16.28	<.001
Ratio of family income to poverty	2.57 ± 1.51	2.66 ± 1.56	2.63 ± 1.51	2.38 ± 1.47	<.001
Gender (%)					<.001
Male	1321 (68.41%)	962 (49.84%)	787 (40.78%)	652 (33.75%)	
Female	610 (31.59%)	968 (50.16%)	1143 (59.22%)	1280 (66.25%)	
Race (%)					<.001
Mexican American	148 (7.66%)	244 (12.64%)	289 (14.97%)	251 (12.99%)	
Other Hispanic	155 (8.03%)	219 (11.35%)	233 (12.07%)	199 (10.30%)	
Non-Hispanic White	718 (37.18%)	680 (35.23%)	673 (34.87%)	660 (34.16%)	
Non-Hispanic Black	483 (25.01%)	431 (22.33%)	461 (23.89%)	584 (30.23%)	
Other race	427 (22.11%)	356 (18.45%)	274 (14.20%)	238 (12.32%)	
Education level (%)					<.001
Less than high school	835 (43.24%)	795 (41.19%)	839 (43.47%)	815 (42.18%)	
High school	569 (29.47%)	596 (30.88%)	639 (33.11%)	730 (37.78%)	
More than high school	527 (27.29%)	539 (27.93%)	452 (23.42%)	387 (20.03%)	
Marital status (%)					<.001
Married/Living with partner	1097 (56.81%)	1202 (62.28%)	1150 (59.59%)	1063 (55.02%)	
Widowed/Divorced/Separated	375 (19.42%)	437 (22.64%)	474 (24.56%)	440 (22.77%)	
Never married	459 (23.77%)	291 (15.08%)	306 (15.85%)	429 (22.20%)	
Drink (%)					.117
Yes	1782 (92.28%)	1747 (90.52%)	1760 (91.19%)	1784 (92.34%)	
No	149 (7.72%)	183 (9.48%)	170 (8.81%)	148 (7.66%)	
Hyperlipidemia (%)					<.001
Yes	528 (27.34%)	761 (39.43%)	783 (40.57%)	690 (35.71%)	
No	1403 (72.66%)	1169 (60.57%)	1147 (59.43%)	1242 (64.29%)	
Diabetes (%)					<.001
Yes	103 (5.33%)	245 (12.69%)	393 (20.36%)	446 (23.08%)	
No	1781 (92.23%)	1655 (85.75%)	1452 (75.23%)	1416 (73.29%)	
Borderline	47 (2.43%)	30 (1.55%)	85 (4.40%)	70 (3.62%)	
Kidney stones (%)					.009
Yes	148 (7.66%)	185 (9.59%)	203 (10.52%)	201 (10.40%)	
No	1783 (92.34%)	1745 (90.41%)	1727 (89.48%)	1731 (89.60%)	
Congestive heart failure (%)					.003
Yes	61 (3.16%)	50 (2.59%)	87 (4.51%)	83 (4.30%)	
No	1870 (96.84%)	1880 (97.41%)	1843 (95.49%)	1849 (95.70%)	
Coronary heart disease (%)					.016
Yes	80 (4.14%)	78 (4.04%)	110 (5.70%)	73 (3.78%)	
No	1851 (95.86%)	1852 (95.96%)	1820 (94.30%)	1859 (96.22%)	
Smoking status (%)					<.001
Yes	908 (47.02%)	770 (39.90%)	752 (38.96%)	798 (41.30%)	
No	1023 (52.98%)	1160 (60.10%)	1178 (61.04%)	1134 (58.70%)	

HSI = hepatic steatosis index

### 3.2. The correlation between HSI and hypertension

In the continuous model, each unit increase in HSI was associated with a 4% higher risk of hypertension after full adjustment (OR = 1.04, 95% CI: 1.03–1.05, *P* < .0001). A significant dose-response relationship was observed across HSI quartiles, with the highest quartile (Q4) was associated with 2.65-fold higher risk compared to Q1 (95% CI: 2.23–3.15, *P* < .0001), in the fully-adjusted model (Table 2).Strong linear trends were confirmed through quartile analyses (*P*-trend < .0001 in all models). The risk gradient remained robust after sequential adjustments: Model 1 (unadjusted): Q4 OR = 2.22 (1.94–2.54), Model 2 (demographic-adjusted): Q4 OR = 3.52 (2.99–4.13), Model 3 (fully-adjusted): Q4 OR = 2.65 (2.23–3.15).

**Table 2:** Association between HSI quartiles and hypertension risk.

HSI	Hypertension OR (95% CI)		
Ex.posure	Model 1	Model 2	Model 3
HSI continues (n = 7723)	1.03 (1.02–1.04) < 0.0001	1.05 (1.05–1.06) < 0.0001	1.04 (1.03–1.05) < 0.0001
Quartile			
Q1 (n = 1931)	1.0	1.0	1.0
Q2 (n = 1930)	1.56 (1.36–1.79) < 0.0001	1.70 (1.45–1.98) < 0.0001	1.49 (1.27–1.76) < 0.0001
Q3 (n = 1930)	1.99 (1.74–2.28) < 0.0001	2.44 (2.09–2.86) < 0.0001	1.93 (1.63–2.28) < 0.0001
Q4 (n = 1932)	2.22 (1.94–2.54) < 0.0001	3.52 (2.99–4.13) < 0.0001	2.65 (2.23–3.15) < 0.0001
*P* for trend	<.0001	<.0001	<.0001

Model 1: Unadjusted; Model 2: Adjusted for gender, sex, and race; Model 3: Adjusted for gender, sex, and race, income-to-poverty ratio, education level, marital status, drink, smoking status, hypercholesterolemia, diabetes, kidney stones, coronary heart disease, and congestive heart failure. Results were expressed as odds ratios (ORs) with 95% confidence intervals (CIs).

CI = confidence interval, HSI = hepatic steatosis index, OR = odds ratio.

### 3.3. Nonlinear relationships

**Figure 2.**
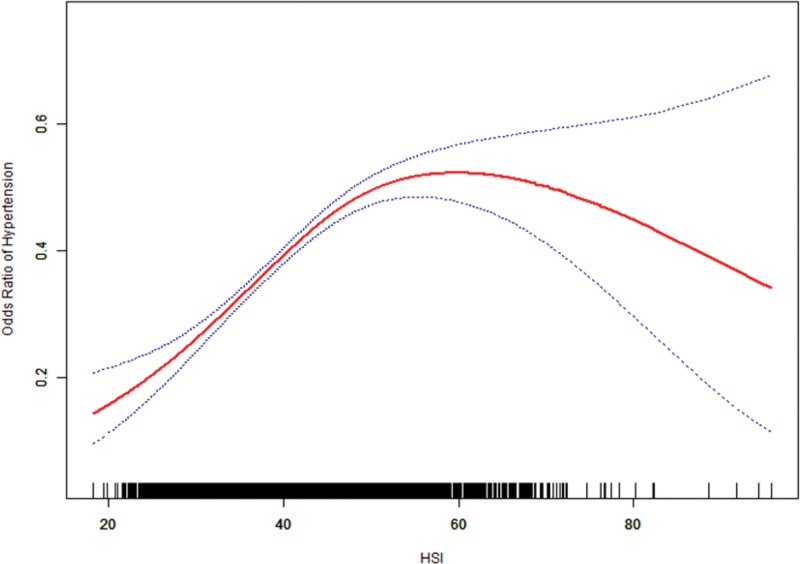
Smooth curve fitting for HSI and hypertension. HSI = hepatic steatosis index.

Our analysis revealed a significant nonlinear association between HSI and hypertension risk (Fig. 2), The threshold analysis demonstrated superior model fit for the 2-part logistic regression over the standard linear model (log-likelihood ratio *P* < .001), identifying a critical inflection point at HSI = 50.33. Below this threshold, each unit HSI was associated with a 6% higher risk of hypertension (OR = 1.06, 95% CI: 1.05–1.07; *P* < .0001); above the threshold, no significant association was observed (OR = 0.99, 95% CI: 0.97–1.01; *P* = .181). These findings contrast with the linear model’s uniform 4% risk elevation per HSI unit (OR = 1.04, 95% CI: 1.03–1.05; *P* < .0001), revealing that the association between hepatic steatosis (as reflected by HSI) and hypertension risk appeared to stabilize beyond HSI ≥ 50.33 (Table 3).

**Table 3:** Threshold effect analysis of HSI on hypertension.

Models	Hypertension OR (95% CI)
Standard linear model	1.04 (1.03–1.05) <0.0001
Two-part logistic regression model	
Inflection point (K)	50.33
HSI < 50.33	1.06 (1.05–1.07) <0.0001
HSI ≥ 50.33	0.99 (0.97–1.01) 0.1806
Log-likelihood ratio	<0.001

Adjusted for gender, sex, and race, income-to-poverty ratio, education level, marital status, drink, smoking status, hypercholesterolemia, diabetes, kidney stones, coronary heart disease, and congestive heart failure.

CI = confidence interval, HSI = hepatic steatosis index, OR = odds ratio.

Adjusted for demographic factors (age, sex, and race/ethnicity); further adjusted for socioeconomic status (income-to-poverty ratio, educational attainment, marital status), lifestyle factors (alcohol consumption, smoking status), and comorbidities (hypercholesterolemia, diabetes mellitus, kidney stones, coronary heart disease, and congestive heart failure).

### 3.4. Subgroup analysis

The stratified analyses demonstrated significant heterogeneity in the association between HSI and hypertension (Table 4). Notable interaction effects (*P*_interaction_ < 0.05) were observed across sex (*P* < .0001), age (*P* = .0157), diabetes (*P* = .0126), congestive heart failure (*P* = .0253), coronary heart disease (*P* = .0056), and smoking status (*P* = .0259). Males exhibited stronger associations (β = 1.06, 95% CI: 1.05–1.07) than females (β = 1.02, 95% CI: 1.01–1.03), while individuals aged ≥ 60 years (β = 1.05 vs <40 years: β = 1.03) and those with diabetes (β = 1.06 vs non-diabetic: β = 1.04) showed heightened susceptibility. Cardiovascular comorbidities amplified the risk, with congestive heart failure (β = 1.09) and coronary heart disease (β = 1.11) subgroups demonstrating markedly elevated effects. Conversely, nonsmokers had higher risk increments (β = 1.05) compared to smokers (β = 1.03). No significant effect modification was observed for hypercholesterolemia (*P* = .9918), kidney stones (*P* = .6842), or alcohol use (*P* = .7589). Intriguingly, borderline diabetes showed no association (β = 0.99, *P* = .735).

**Table 4:** Subgroup analysis of HSI-hypertension association by clinical characteristics.

Subgroup	N	OR and 95% CI	*P*	*P* interaction
Gender				<.0001
Male	3722	1.06 (1.05, 1.07)	<.0001	
Female	4001	1.02 (1.01, 1.03)	<.0001	
Age				.0157
<40	2470	1.03 (1.02, 1.04)	<.0001	
40–60	2649	1.03 (1.02, 1.04)	<.0001	
60+	2604	1.05 (1.04, 1.06)	<.0001	
Diabetes				.0126
Yes	1187	1.06 (1.04, 1.08)	<.0001	
No	6304	1.04 (1.03, 1.04)	<.0001	
Borderline	232	0.99 (0.95, 1.04)	.7352	
Hyperlipidemia				.9918
Yes	2762	1.04 (1.03, 1.05)	<.0001	
No	4961	1.04 (1.03, 1.05)	<.0001	
Kidney stones				.6842
Yes	737	1.04 (1.02, 1.07)	.0002	
No	6986	1.04 (1.03, 1.05)	<.0001	
Congestive heart failure				.0253
Yes	281	1.09 (1.04, 1.15)	.0002	
No	7442	1.04 (1.03, 1.04)	<.0001	
Coronary heart disease				.0056
Yes	341	1.11 (1.06, 1.17)	<.0001	
No	7382	1.04 (1.03, 1.04)	<.0001	
Smoke group				.0259
Yes	3228	1.03 (1.02, 1.04)	<.0001	
No	4495	1.05 (1.04, 1.06)	<.0001	
Drink				.7589
Yes	7073	1.04 (1.03, 1.05)	<.0001	
No	650	1.04 (1.02, 1.07)	.001	

Adjusted for gender, sex, and race, income-to-poverty ratio, education level, marital status, drink, smoking status, hypercholesterolemia, diabetes, kidney stones, coronary heart disease, and congestive heart failure.

CI = confidence interval, HSI = hepatic steatosis index, OR = odds ratio.

## 4. Discussion

In this cross-sectional study involving 7723 participants, we explored the association between the HSI and hypertension. Regression analysis confirmed a significant positive association, showing that higher HSI values were linked to an increased risk of hypertension. Further analyses using smoothing curve fitting and threshold effect models supported a nonlinear relationship between HSI and hypertension, with a critical inflection point identified at an HSI of 50.33. Below this threshold, the link between higher HSI and hypertension risk was notably stronger, while above this threshold, the association was no longer significant, suggesting this cutoff may align with distinct pathophysiological stages. Subgroup analyses also revealed that the association between HSI and hypertension varied significantly based on factors, including sex, age, diabetes status, congestive heart failure, coronary heart disease, and smoking status. These findings may be attributed to synergistic pathological effects between hepatic fat accumulation, cardiovascular impairment, and dysregulated glucose metabolism.^[18,19]^

NAFLD has emerged as a predominant global hepatic disorder,^[20]^ exhibiting strong epidemiological links to obesity, insulin resistance, and cardiometabolic dysfunction.^[21]^ Beyond its hepatic manifestations, NAFLD is strongly associated with systemic metabolic dysregulation and alterations blood pressure homeostasis through multiple pathways,^[22]^ including visceral adiposity,^[23]^ oxidative stress,^[24]^ excessive proinflammatory cytokine secretion,^[25]^ and endothelial dysfunction,^[26]^ all established correlates of hypertension.^[22]^ Although the NAFLD-hypertension association is well-documented, prior studies predominantly relied on advanced diagnostic modalities with significant limitations: liver biopsy (the gold standard for steatosis assessment) is constrained by invasiveness and sampling variability; ultrasound-based imaging exhibits limited sensitivity for mild steatosis and operator-dependent reproducibility; while transient elastography and MRI-PDFF face accessibility barriers due to cost and technical complexity, particularly in resource-limited settings.^[27,28]^ These challenges underscore the critical need for noninvasive alternatives such as the HSI to facilitate large-scale assessment and clinical implementation.^[29]^

In addition to evaluating NAFLD, multiple studies have revealed significant associations between the HSI and various diseases. A large-scale real-world cohort study from China showed that in patients with comorbid obstructive sleep apnea and hypertension, each 1-unit increase in HSI was associated with a 43% higher risk of major adverse cardiovascular and cerebrovascular events, a 38% higher risk of cardiac events, and a 51% higher risk of cerebrovascular events.^[30]^ Another longitudinal study from Japan demonstrated a significant association between elevated HSI and the likelihood of developing type 2 diabetes mellitus in individuals with normal blood glucose levels.^[31]^ A cross-sectional study in China indicated that elevated HSI is an independent risk factor for endometrial cancer.^[32]^ Furthermore, a prospective cohort study involving 39,114 pregnant women in China showed that the risk of gestational hypertension significantly increased with each quartile elevation of HSI. Compared with women aged 35 years or older, HSI had a relatively stronger predictive value for gestational hypertension and preeclampsia in pregnant women under 35 years old.^[11]^ A comprehensive study conducted in Japan involving 94,893 participants investigated the association between HSI and chronic kidney disease (CKD), revealing a clear dose-response relationship and indicating that HSI is associated with CKD in middle-aged Japanese individuals.^[33]^ These evidences, mutually corroborating the conclusions of this study, collectively establish the central role of HSI in the network of chronic metabolic diseases.

The role of HSI in the pathological process of hypertension involves complex mechanisms across multiple dimensions, with insulin resistance and inflammatory responses regarded as key driving factors.^[14,16,34]^ As a hallmark pathological feature of NAFLD, insulin resistance is often accompanied by increased oxidative stress and is linked to pathological progression of NAFLD by activating hepatic lipid synthesis pathways and potentially involving activation of inflammatory cascades.^[35,36]^ From the perspective of blood pressure regulation, insulin resistance is associated with impaired renal sodium and water handling, which coincides with increased peripheral blood volume and elevated blood pressure levels.^[37]^

Furthermore, our study found an established significant positive association between the HSI and hypertension, providing critical evidence for HSI as a potential marker in assessing hypertension risk investigation in longitudinal studies to explore whether interventions targeting hepatic steatosis and insulin resistance could benefit blood pressure management; however, this requires testing in intervention studies. Results of further subgroup analysis show significant interaction effects of this association in populations with coronary heart disease, congestive heart failure, type 2 diabetes mellitus, and smoking status, which could inform health management of specific high-risk groups: For patients with comorbid cardiovascular diseases (such as coronary heart disease and congestive heart failure), monitoring HSI alongside blood pressure might be considered to provide a more comprehensive risk profile; For patients with type 2 diabetes mellitus, attention to both HSI and blood glucose levels may be warranted, given their interconnected associations with metabolic dysregulation and hypertension; For smoking populations, the findings highlight the combined impact of smoking and altered hepatic lipid metabolism on hypertension risk. This suggests that interventions targeting both smoking cessation and metabolic health may be synergistic, although this potential synergy requires confirmation.

The strengths of this study lie in the utilization of data from a nationally representative database, which enhances the credibility of the results, the adoption of a standardized statistical analysis framework, and the provision of stratified disease prevention and control strategies for specific populations through further subgroup analysis. Meanwhile, this study also has several limitations. First, as a cross-sectional analysis, it cannot determine causal relationships, and reverse causality may exist. Second, self-reported hypertension may have biases from hypertensive patients who have not sought drug treatment and those whose blood pressure has not been measured. It is also impossible to perform subgroup analysis based on blood pressure categories, and confounders such as C-reactive protein and medication use were not excluded. Most importantly, our study did not use NHANES sampling weights, which may affect the representativeness of the results for the adult population in the United States. Therefore, more rigorous prospective cohort studies or randomized controlled trials are needed in the future to determine the causal relationship between HSI and hypertension, minimize the interference of confounding factors, and further verify the reliability of the results of this study.

## 5. Conclusion

In conclusion, our study found a substantial positive relationship between HSI and hypertension risk. This connection exhibits nonlinearity and population heterogeneity. The threshold and subgroup findings can guide future risk stratification techniques based on gender, age, comorbidities, and lifestyle factors.

## Acknowledgments

A special thanks to all of the NHANES participants who freely gave their time to make this and other studies possible.

## Author contributions

**Conceptualization:** Zihao Zhao.

**Data curation:** Zihao Zhao, Weizhong Huangfu.

**Formal analysis:** Zihao Zhao.

**Investigation:** Zihao Zhao.

**Methodology:** Zihao Zhao.

**Resources:** Zihao Zhao.

**Software:** Zihao Zhao.

**Supervision:** Yuhong Ma.

**Validation:** Yuhong Ma.

**Visualization:** Zihao Zhao.

**Writing – original draft:** Zihao Zhao.

**Writing – review & editing:** Weizhong Huangfu.
